# Ovulation induction with minimal dose of follitropin alfa: a case series study

**DOI:** 10.1186/1477-7827-9-142

**Published:** 2011-10-24

**Authors:** Isidoro Bruna-Catalán, Marco Menabrito

**Affiliations:** 1 Reproduction Unit, Hospital Universitario de Madrid-Montepríncipe, Madrid, Spain; 2 Medical Department, Merck, S.L., an affiliate of Merck KGaA Darmstadt Germany, Madrid, Spain

**Keywords:** Follitropin alfa, ovulation induction, case series study, monofollicular growth, recombinant follicle-stimulating hormone

## Abstract

**Background:**

Gonadotropins are used in ovulation induction (OI) for patients with anovulatory infertility. Pharmacologic OI is associated with risks of ovarian hyperstimulation syndrome and multiple pregnancy. Treatment protocols that minimize these risks by promoting monofollicular development are required. A starting dose of 37.5 IU/day follitropin alfa has been used in OI, particularly among women at high risk of multifollicular development and multiple pregnancy. A retrospective case series study was performed to evaluate rates of monofollicular development and singleton pregnancy following standard treatment with 37.5 IU/day follitropin alfa.

**Methods:**

Spanish centers that had performed at least five OI cycles during 2008 using 37.5 IU/day follitropin alfa as a starting dose were invited to participate. Data could be provided from any cycle performed in 2008 (up to a maximum of 12 consecutive cycles per site). Case report forms were collected during April-November 2009 and reviewed centrally. Descriptive statistics were obtained from all cases, and follicular development and clinical pregnancy rates assessed. Potential associations of age and body mass index with follicular development and clinical pregnancy were assessed using univariate correlation analyses.

**Results:**

Thirty centers provided data on 316 cycles of OI using a starting dose of 37.5 IU/day follitropin alfa. Polycystic ovary syndrome was the cause of anovulatory infertility in 217 (68.7%) cases. Follitropin alfa at 37.5 IU/day was sufficient to achieve ovarian stimulation in 230 (72.8%) cycles. A single follicle ≥16 mm in diameter developed in 193 cycles (61.1%; 95% confidence interval [CI] 55.7-66.4%). Seventy-eight women (24.7%; 95% CI 19.9-29.5%) became pregnant: 94.9% singleton and 5.1% twin pregnancies. Fourteen started cycles (4.4%) were cancelled, mainly due to poor response. Univariate correlation analyses detected weak associations.

**Conclusions:**

Monofollicular growth rate was comparable with optimal rates reported elsewhere and the pregnancy rate exceeded that in other studies of OI using gonadotropins. A starting dose of 37.5 IU/day follitropin alfa is an effective option in selected cases to prevent ovarian hyper-response without loss of efficacy. The analysis could not identify a single selection criterion for individuals who would benefit from this treatment approach; this merits further investigation in prospective studies.

## Background

Exogenous follicle-stimulating hormone (FSH) is the most common treatment for chronic anovulatory infertility. Pharmacologic ovulation induction (OI) results in a pregnancy rate of 10-20% per cycle [1]. However, treatment with exogenous gonadotropins carries a risk of multifollicular development, leading to multiple pregnancy in 5-20% of cycles. A 50% increase in twin birth rates has been observed over the last three decades, and high-order multiple birth rates have increased even more dramatically. This is linked to the increased use of gonadotropins to induce ovulation [1,2]. Furthermore, precautionary cancellation of cycles may be required when more than three follicles ≥16 mm in diameter develop [3,4]. Indeed, 5-10% of started cycles are cancelled to prevent ovarian hyperstimulation syndrome (OHSS) [5-9].

Various therapeutic strategies have been attempted to limit the growth and development of multiple preovulatory ovarian follicles [6,7,10-12]. Efforts have been made to modulate ovarian response to gonadotropins by concurrent treatment with anti-estrogens, aromatase inhibitors, and gonadotropin-releasing hormone analogs to reduce the dose of gonadotropin required [12-14], and by directly lowering the standard doses of gonadotropins administered [15-17]. These approaches have had limited success and multiple follicular development, multiple pregnancy, and OHSS still remain as major, unresolved issues in OI.

In recent years, interest has been growing in the use of ultra-low-dose FSH regimens in OI for women with polycystic ovary syndrome (PCOS). In one study in PCOS, OI using a chronic low-dose FSH regimen resulted in a low multiple (twin) pregnancy rate (5%) and no reported cases of OHSS without compromising the clinical pregnancy rate (29% per started cycle) [18]. By contrast, a conventional treatment protocol resulted in a multiple pregnancy rate of almost 36% and a severe OHSS rate of 4.6% [19].

Ovarian stimulation with low-dose gonadotropins is based on the concept of the 'FSH threshold' proposed many years ago by Brown, who suggested that a threshold level of FSH must be reached to achieve follicular development [20]. A slight elevation in plasma FSH concentration (10-30% above the threshold) is sufficient to stimulate normal follicular development, whereas further elevation may cause hyper-stimulation.

Conventional OI regimens use supraphysiological doses of FSH, whereas low-dose regimens aim to develop an initial cohort of follicles without exceeding the FSH threshold. Low-dose protocols usually involve administration of a starting dose of 50-75 IU FSH for 7-14 days; if necessary, the dose is increased by 50% until follicular development begins [21]. Once a dominant follicle is identified, FSH treatment is maintained until the criteria for human chorionic gonadotropin (hCG) administration are fulfilled. Ovulation is reported to be achieved in approximately 70% of low-dose FSH treatment cycles [22-24]. However, a recent consensus statement concluded that, although low-dose FSH is effective in OI for women with PCOS, further refinements to treatment protocols are required to optimize outcomes [25].

Clinicians understand that the use of gentler stimulation regimens could reduce the incidence of high-order multiple pregnancy, but would increase the rate of cycle cancellation due to poor ovarian response and reduce pregnancy rates (with the associated human and financial cost implications). Nevertheless, practitioners do identify individuals who are at particular risk of multiple pregnancy. For these patients, the use of ultra-low-doses (37.5 IU/day) of follitropin alfa, a recombinant FSH, is considered to be a safer approach for achieving pregnancy while limiting the chance of multiple pregnancy.

Because of the sporadic nature of this clinical practice, no formal study has been published. Therefore, the outcomes remain unknown, filed only in the archives of individual medical centers. In this pilot study, we collected existing single case records to create a case series. The main aim of the study was to determine the rate of monofollicular development with a starting dose of 37.5 IU/day follitropin alfa in OI. Additionally, patient characteristics, the total dose of follitropin alfa required, and rates of singleton and multiple pregnancy and cycle cancellation were evaluated. The results of this study may provide the basis for a follow-up, prospective clinical trial.

## Methods

### Case series and scope

This retrospective case series study was based on data from case reports of women with anovulatory infertility (mainly due to PCOS) who were treated with follitropin alfa (GONAL-f^®^, Merck Serono S.A. - Geneva, Switzerland, a branch of Merck Serono S.A., Coinsins, Switzerland, an affiliate of Merck KGaA, Darmstadt, Germany) at a starting dose of 37.5 IU/day to induce ovulation according to standard practice criteria. Any Spanish infertility center that had conducted at least five such OI cycles during 2008 was eligible to participate. In order to standardize and balance participation, data from a maximum of 12 consecutive cases were allowed per site. The study was restricted to cycles of OI conducted during 2008 (although cycles performed up to April 1, 2009 were considered as conducted in the previous year).

### Data collection

Case report forms (CRFs) were provided to centers willing to participate and able to provide at least five suitable case reports. Each CRF included sections relating to baseline characteristics, infertility history, follitropin alfa dosage, duration of ovarian stimulation, follicular development, and the number of gestational sacs. Participants completed CRFs with data obtained directly from medical records. As data were coded and dissociated from any personal identifier, neither ethics committee approval nor informed consent was required. Completed CRFs were collected between April and November 2009. Data were reviewed centrally and inconsistencies were resolved before the database was locked on December 20, 2009.

### Outcomes

The primary outcome was the monofollicular development rate, defined as the growth of a single follicle ≥16 mm in diameter on the day of hCG administration. Other outcomes included clinical pregnancy rate at ≥7 weeks gestation and number of gestational sacs (as identified by ultrasound), cycle cancellation rate (with relevant reason), duration of ovarian stimulation, and total dose of follitropin alfa required.

### Data analysis

Descriptive statistics of the study population are presented as mean values with standard deviations for continuous variables and as frequency and percentages for discrete variables. Associated 95% confidence intervals (CI) are provided for the two main variables. Rates were obtained directly as the quotient of the two relevant variables.

The study population was further classified based on age (≤35 or > 35 years) and body mass index (BMI; ≤25 kg/m^2 ^or > 25 kg/m^2^). Differences between these pairs of subgroups were analyzed using the Student's *t*-test, Mann-Whitney U test or Fisher Exact test, as appropriate. Potential associations between follicular development and clinical pregnancy and age, BMI, and total dose of follitropin alfa were assessed through univariate correlation analysis.

Data were evaluated and statistical analyses performed using SPSS (Statistical Package for the Social Sciences).

## Results

Thirty participating centers provided data on 316 cycles in which women received a starting dose of 37.5 IU/day follitropin alfa for OI. This represents 4.4% of the 7176 OI cycles undertaken in these centers within the study period.

Baseline characteristics of the study population are presented in Table 1. The mean age of women in this study was 31.8 years, and the majority of women (262; 82.9%) were ≤35 years of age. Although the duration of infertility was relatively long, exposure to OI treatment was limited, with a mean of only 1.2 previous cycles. PCOS was the cause of anovulatory infertility in more than two-thirds of cases (217; 68.7%). The majority of women (217; 68.7%) had a mean BMI within the normal range (≤25 kg/m^2^; the upper limit of established normal range). Overall, the study population comprised young, slim women with a long history of infertility due to PCOS, but minimal prior exposure to gonadotropins.

**Table 1:** All cases: baseline characteristics, ovarian stimulation, and OI outcome

Characteristic/variable	All cases (n = 316)
Age, years	31.8 (3.6)
BMI, kg/m^2^	23.7 (4.2)
***Etiology***	
PCOS, n (%)	217 (68.7)
Other/unknown, n (%)	99 (31.3)
***Infertility characteristics***	
Duration of infertility, months	27.8 (18.6)
Number of previous OI cycles	1.2 (1.2)
***Ovarian stimulation***	
Starting daily dose, IU	37.5 (0.0)
Cycles that completed at 37.5 IU, n (%)	230 (72.8)
Cycles that required a dose increase, n (%)	86 (27.2)
Final daily dose, IU	49.9 (24.1)
Total dose of r-hFSH, IU	517 (410)
Duration of stimulation, days	11.0 (5.1)
Cycles cancelled, n (%)	14 (4.4)
Poor response, n (%)	10 (3.0)
Risk of OHSS, n (%)	4 (1.3)
***Follicular development***	
Number of follicles ≥16 mm in diameter	1.4 (0.7)
Cycles resulting in 0 follicles, n (%)	9 (2.8)
Cycles resulting in 1 follicle, n (%)	193 (61.1)
Cycles resulting in 2 follicles, n (%)	84 (26.6)
Cycles resulting in ≥3 follicles, n (%)	29 (9.2)
Cycles with no data, n (%)	1 (0.3)
***OI outcome***	
Clinical pregnancy, * n (%)	78 (24.7)
Singleton pregnancy, n (%)	74 (94.9)
Twin pregnancy, n (%)	4 (5.1)

Values are mean (SD) unless otherwise indicated.

*Defined as gestation ≥7 weeks.

BMI, body mass index; OHSS, ovarian hyperstimulation syndrome; OI, ovulation induction; PCOS, polycystic ovary syndrome; r-hFSH, recombinant human follicle-stimulating hormone; SD, standard deviation.

A starting dose of 37.5 IU/day follitropin alfa was sufficient to achieve ovarian stimulation in 230 (72.8%) cycles, while the remainder (86; 27.2%) required only small incremental dose increases (as reflected by a mean final daily dose of 49.9 IU). The mean duration of ovarian stimulation was 11.0 days and a mean total dose of 517 IU follitropin alfa was required. A single follicle ≥16 mm in diameter developed in the majority of cycles (61.1%; 95% CI 55.7-66.4%) and the pooled rate of mono- or bifollicular development approached 90%. The cancellation rate was 4.4%, and was mainly due to a poor ovarian response. Clinical pregnancy was achieved in 24.7% (95% CI 19.9-29.5%) of started cycles. Most pregnancies were singletons (94.9%); only four twin pregnancies occurred and there were no high-order multiple pregnancies.

Outcomes among women aged ≤35 years and those aged > 35 years were compared, as a cut-off of 35 years is generally agreed to designate advanced reproductive age for assisted reproduction purposes. As shown in Table 2, there was a difference in mean age of 6.4 years between the subgroups (30.7 vs 37.1 years; p = 0.0001). The only other significant difference between subgroups was that the mean number of previous OI cycles in the younger subgroup was double that of the older subgroup (1.9 vs 0.8; p = 0.02). A trend toward an increased pregnancy rate was also observed in the younger versus the older subgroup, despite a higher cycle cancellation rate. In addition, the four cycles that were cancelled because of a risk of OHSS, as well as the four twin pregnancies, all occurred in the subgroup of women aged ≤35 years.

**Table 2:** Subgroup analysis of women aged ≤35 years versus > 35 years

Characteristic/variable	≤35 years (n = 262)	> 35 years (n = 54)	p value
Age, years	30.7 (2.9)	37.1 (1.1)	0.0001
BMI, kg/m^2^	23.5 (4.7)	24.6 (3.6)	NS
PCOS, n (%)	182 (69.5)	35 (64.8)	NS
Other/unknown etiology, n (%)	80 (30.5)	19 (35.2)	NS
Duration of infertility, months	27.6 (17.9)	27.8 (21.0)	NS
Number of previous OI cycles	1.9 (1.3)	0.8 (0.8)	0.02
Duration of stimulation, days	11.1 (5.2)	10.6 (4.9)	NS
Total dose of r-hFSH, IU	525 (425)	486 (333)	NS
Cycles cancelled, n (%)	13 (5.0)	1 (1.9)	NS
Poor response, n (%)	9 (3.4)	1 (1.9)	NS
OHSS risk, n (%)	4 (1.5)	0 (0)	NS
Number of follicles ≥16 mm in diameter	1.3 (0.6)	1.5 (0.7)	NS
Clinical pregnancy, * n (%)	67 (25.6)	11 (20.4)	NS
Singleton pregnancy, n (%)	63 (94.0)	11 (100)	NS
Twin pregnancy, n (%)	4 (6.0)	0 (0)	NS

Values are mean (SD) unless otherwise indicated.

*Defined as gestation ≥7 weeks.

BMI, body mass index; NS, not significant; OHSS, ovarian hyperstimulation syndrome; OI, ovulation induction; PCOS, polycystic ovary syndrome; r-hFSH, recombinant human follicle-stimulating hormone; SD, standard deviation.

Outcomes among women with a BMI of ≤25 kg/m^2 ^and > 25 kg/m^2 ^were also compared, as being overweight negatively affects the ovarian response to OI. A significant difference of 7.5 kg/m^2 ^in mean BMI score was found between the BMI ≤25 kg/m^2 ^and > 25 kg/m^2 ^subgroups (p = 0.0001; Table 3). This difference reflected a mean difference of 19 kg in body weight. As expected, most of the cases (89%) in the BMI > 25 kg/m^2 ^subgroup presented with PCOS versus 60% of cases in the BMI ≤25 kg/m^2 ^subgroup (p = 0.0001). Ovarian resistance to stimulation is well recognized in women with excess body weight. This effect resulted in a significantly longer mean duration of stimulation (2.1 days; p = 0.001) and a higher mean follitropin alfa requirement (32 IU per cycle; p = 0.005) in the high versus low BMI subgroup. All other variables were similar in both subgroups, although the pregnancy rate was slightly higher (24.9% vs 20.7%) in the low versus high BMI subgroup.

**Table 3:** Subgroup analysis of women with BMI ≤25 kg/m^2 ^vs > 25 kg/m^2^

Characteristic/variable	≤25 kg/m^2 ^(n = 217)	> 25 kg/m^2 ^(n = 91)	p value
Age, years	31.6 (3.69)	32.3 (3.7)	NS
BMI, kg/m^2^	21.5 (2.1)	29.0 (3.3)	0.0001
PCOS, n (%)	131 (60.4)	81 (89.0)	0.0001
Other/unknown etiology, n (%)	84 (38.7)	10 (11.0)	0.0001
Duration of infertility, months	29.0 (3.3)	28.0 (18.8)	NS
Number of previous OI cycles	1.2 (1.2)	1.2 (1.2)	NS
Duration of stimulation, days	10.4 (5.5)	12.5 (4.0)	0.001
Total dose of r-hFSH, IU	574 (417)	606 (356)	0.005
Cycles cancelled, n (%)	11 (5.1)	3 (3.3)	NS
Poor response, n (%)	8 (3.7)	2 (2.2)	NS
OHSS risk, n (%)	3 (1.4)	1 (1.1)	NS
Number of follicles ≥16 mm in diameter	1.4 (0.6)	1.4 (0.3)	NS
Clinical pregnancy, * n (%)	54 (24.9)	19 (20.7)	NS
Singleton pregnancy, n (%)	52 (96.3)	17 (89.5)	NS
Twin pregnancy, n (%)	2 (3.7)	2 (10.5)	NS

BMI data not recorded in eight cases, so n = 308.

Values are mean (SD) unless otherwise indicated.

*Defined as gestation ≥7 weeks.

BMI, body mass index; NS, not significant; OHSS, ovarian hyperstimulation syndrome; OI, ovulation induction; PCOS, polycystic ovary syndrome; r-hFSH, recombinant human follicle-stimulating hormone; SD, standard deviation.

Univariate correlation analyses were used to identify potential associations between follicles ≥16 mm in diameter or clinical pregnancy with age, BMI, or total dose of follitropin alfa. Weak positive associations were found between all pairs of variables analyzed; however, none were statistically significant.

## Discussion

The aim of this case series study was to evaluate the effectiveness of 37.5 IU/day follitropin alfa as a starting dose in OI for women with anovulatory infertility. No data on this therapeutic approach were previously available. Because of the reduced risk of ovarian hyper-response associated with this low dose, we conducted a case series study from existing single case reports. We showed that the use of 37.5 IU/day follitropin alfa can prevent multifollicular growth among women at risk of ovarian hyper-response and multiple pregnancy.

This ultra-low-dose approach (37.5 IU/day follitropin alfa) led to the development of a single follicle ≥16 mm in diameter in 61.1% and clinical pregnancy in 24.7% of started cycles. Indeed, rates of both monofollicular development and clinical pregnancy are higher than those reported in OI using a standard daily dose of 75 IU FSH. The total dose of follitropin alfa required was 517 IU and mean duration of ovarian stimulation was 11.0 days, indicating that minimal dose adjustment was needed. Notably, despite the ultra-low-dose regimen, only 14 (4.4%) cycles were cancelled and almost all were due to poor ovarian response. Only 4 (1.3%) cycles were cancelled because of a risk of OHSS (based on the number of follicles on ultrasound), thus fulfilling a major goal in OI. Both the lower total dose of follitropin alfa required and the reduction in risk of OHSS could improve the patients' experience of OI.

Data from a recently published large, prospective, observational study provide a broad representation of the use of recombinant human FSH (r-hFSH) in OI in French clinical practice (n = 1398) [26]. Most patients receiving treatment (91%) had WHO Group II anovulatory infertility and, of these, 35% had a diagnosis of PCOS. A starting dose of 75 IU/day r-hFSH, without adjustment, stimulated the development of 1.3 ± 1.5 follicles ≥15 mm in diameter. The clinical pregnancy rate was 18% per started cycle; 16% of these were multiple pregnancies (14% twins and 2% high-order multiple pregnancies). The cycle cancellation rate was 11%, and was mainly due to ovarian hyper-response. The rates of multiple pregnancy and cycle cancellation due to hyper-response indicate that there is still scope for improvement in OI treatment protocols, and supports the search for milder treatment strategies to optimize outcomes.

The use of low gonadotropin doses to induce the maturation of a single preovulatory follicle seems to represent the most realistic therapeutic approach to OI at present. A starting dose of 50 IU/day r-hFSH was evaluated in a large, observational study in OI in 88 Spanish clinics [17]. Data were included on 343 women with anovulatory infertility, of whom two-thirds (231; 67%) had ultrasound evidence of PCOS. Of 945 cycles started, 817 cycles were completed, resulting in a 14% cancellation rate (due to spontaneous ovulation or ovarian hyper-response). The monofollicular development rate was 53% (501/945) per started cycle. A total of 136 clinical pregnancies occurred (14% per started cycle), with eight twin and no high-order multiple pregnancies. Sixty-four (6.8%) cases of OHSS were recorded but none required hospitalization. The median daily dose of r-hFSH in all cycles was the starting dose of 50 IU. This study supports the use of 50 IU/day r-hFSH in OI for anovulatory infertility and the possibility of even lower doses for some patients.

Two low-dose, step-up FSH regimens for OI were compared in a trial at 18 European and Canadian centers [11]. Patients with WHO Group II anovulatory infertility (n = 158) received 50 IU/day r-hFSH for 7 days and then the daily dose was increased by increments of 25 or 50 IU r-hFSH per week. The monofollicular development rate in the lower dose group was almost twice that of the higher dose group (41% vs 22%, respectively). The ongoing pregnancy rate was 20% and 13% in the 25 IU and 50 IU dose groups, respectively. The cancellation rate due to ovarian hyper-response was fourfold greater in the higher versus lower dose group (21% vs 5%, respectively), whereas the cancellation rate due to poor ovarian response was 5% in both groups.

The ability to accurately predict the FSH threshold would, theoretically, enable clinicians to prescribe the lowest effective dose to induce monofollicular development for each individual patient. Eight variables were found to be predictive of the FSH threshold dose in OI in a multivariate analysis of data for women with anovulatory infertility [27]. Three variables (BMI, spontaneous menstrual cycle history, and ovarian volume) were found to be significant, independent predictors of the FSH threshold dose, and a simple nomogram based on these predictive factors was constructed. The daily doses used in this step-up protocol ranged from 75 to 187.5 IU FSH and were, therefore, higher than the starting daily dose of 37.5 IU evaluated in the current case series study.

Although the clinicians participating in the current study used criteria to identify cases suitable for an ultra-low-dose follitropin alfa regimen, our analysis of data from this case series failed to identify single variables that were associated with a monofollicular response. Therefore, we were unable to identify women who would be most likely to benefit from this therapeutic approach. The complexity of the criteria and the circumstances that clinicians take into account when choosing treatment protocols could not be captured in this study, and requires further investigation.

We acknowledge that observational studies, such as this case series, have a number of limitations. Our study was uncontrolled, there was a potential risk of bias in the selection and reporting of cases, and data collection ultimately depended on the availability and accuracy of medical records. For example, data on live births would have been useful but were not readily available from infertility center records. However, efforts were made to address these issues and ensure that valid information was obtained. For example, a short set of universally available data were collected from each case to minimize any site-related discrepancies in standard practice. In addition, no fewer than five and no more than 12 consecutive case reports were provided by each center. Furthermore, the study window was limited to a single year (2008) to minimize potential changes in practice. Thus, we believe that our findings provide valuable information on the use of follitropin alfa at a starting dose of 37.5 IU in OI.

The results of this case series study support the use of ultra-low-dose follitropin alfa (37.5 IU/day) in OI for women with anovulatory infertility. Infertile women who wish to become pregnant often overlook the risks of multiple pregnancy or OHSS. Experienced clinicians can effectively advise individuals who are at particularly high risk of OHSS on the use of low-dose therapy to maximize the likelihood of pregnancy while avoiding high-order multiple pregnancy. The potential benefits of ultra-low-dose follitropin alfa treatment (37.5 IU/day) merits further investigation in prospective studies to identify patients who could gain the greatest benefit from this therapeutic approach.

## Competing interests

IB-C has received consultation fees from Merck S.L., Madrid Spain (an affiliate of Merck KGaA, Darmstadt, Germany). MM is an employee of Merck S.L., Madrid, Spain (an affiliate of Merck KGaA, Darmstadt, Germany).

## Authors' contributions

IB-C contributed to the design of the protocol and critically reviewed drafts of the manuscript. MM carried out the statistical analysis and helped to write the first draft of the manuscript. Both authors read and approved the final version of the manuscript.
